# Retraction Note to: Stromal vascular fraction cells for the treatment of critical limb ischemia: a pilot study

**DOI:** 10.1186/s12967-020-02266-3

**Published:** 2020-02-18

**Authors:** Adas Darinskas, Mindaugas Paskevicius, Gintaras Apanavicius, Gintaris Vilkevicius, Liutauras Labanauskas, Thomas E. Ichim, Rytis Rimdeika

**Affiliations:** 1 Laboratory of Immunology, National Cancer Institute, Santariskiu Str. 1, 08660 Vilnius, Lithuania; 2 Department of Vascular Surgery, Vilnius City Clinical Hospital, Antakalnio Str. 57, 10207 Vilnius, Lithuania; 3 Northway Medical and Surgical Center, S.Zukausko Str. 19, 08234 Vilnius, Lithuania; 4 Clinics of Cardiovascular Diseases, Vilnius University, Santariskiu Str. 2, 08661 Vilnius, Lithuania; 5 Department of Plastic and Reconstructive Surgery, Lithuanian University of Health Sciences, Medical Academy, University Clinics of Kaunas, Eiveniu Str. 2, 50009 Kaunas, Lithuania; 6 Immune Advisors LLC, San Diego, CA 92121 USA

## Retraction Note: J Transl Med (2017) 15:143 10.1186/s12967-017-1243-3

The Editor-in-Chief and the publisher have retracted this article [1]. An investigation by the Lithuanian Bioethics Committee concluded that, contrary to the statements in the article, the study described was not conducted in the Vilnius City Clinical Hospital and the Commission of Medical Ethics did not issue any approval for such a study. Additionally, investigation by the State Health Care Accreditation Agency of the Ministry of Health revealed that the records of the patients described in this study could not be located and verified. The Lithuanian Bioethics Committee therefore concluded that the data are unreliable. The Ombudsperson for Academic Ethics and Procedures conducted a follow up investigation and affirmed that the information presented with respect to the location of the study, the ethical approval, and the period of data collection was fictional but was provided as if it was true, i.e. this information was fabricated.

Adas Darinskas and Thomas E. Ichim disagree with this retraction. The other authors did not respond to any correspondence about this retraction.
